# Supplementary material for: Preliminary Results on the Effects of Soybean Isoflavones on Growth Performance and Ruminal Microbiota in Fattening Goats
Source: Animals (Basel). 2024 Apr 15;14(8):1188. doi: 10.3390/ani14081188 (PMC11047704; doi:10.3390/ani14081188)

Effects of soybean isoflavones on growth performance and ruminal microbiota in fattening goats

Yuexin Shao[1†], Junhong Xu[2†], Mengyu Wang[1], Yalun Ren[1], Manhong Wei[3], Bowen Tian[1], Jun Luo[1], Juan J. Loor[3*], Huaiping Shi[1*],

[1]College of Animal Science and Technology, Northwest A&F University, Yangling, Shaanxi, 712100, China; shaoyuexin0423@163.com (YS); wmy914624@163.com(MW);yalunren2024@163.com(YR); tianbowen310@163.com (BT)

[2]Weinan Agricultural Products Quality and Safety Inspection and Testing Center, Weinan Shaanxi, 714000, China; xujunhong000@soho.com(JX);

[3]College of Animal Engineering, Yangling Vocational & Technical College, Yangling, 712100, China; nwsuafwmh@163.com(MW);

[4]Department of Animal Sciences and Division of Nutritional Sciences, University of Illinois, Urbana, Illinois 61801, United States of America;

*Correspondence: huaipingshi@nwafu.edu.cn(HS) Tel.: +86-029- 87092102; jloor@illinois.edu(Juan J.Loor)

† Equally contributing authors.

Supplemental tables

Supplemental Table S1 Composition and nutrient content of the basal diet (air-dry basis) %

Items	Content
Corn	16.50
Soybean meal	7.50
Wheat bran	3.60
Rapeseed meal	0.90
$CaHPO_4$	0.45
NaCl	0.45
Premix[1)]	0.60
Alfalfa hay	21.00
Corn silage	49.00
Total	100.00
Nutrient levels[2)]	
ME (MJ/kg) [3)]	10.94
OM	93.34
CP	16.67
EE	3.13
NDF	22.86
ADF	7.54

1) The premix included the following per kg of diets: Vitamin A 170 KIU, Vitamin D3 3.40 KIU, Mn (as manganese sulfate) 350 mg, Zn (as zinc sulfate) 586 mg, Cu (as copper sulfate) 256 mg, Fe (as ferrous sulfate) 765 mg.
2) CP, EE, OM, NDF and ADF were measured values.
3) ME was calculated according to NRC (2007) [44].

Supplemental Table S2 Certificate of analysis of isoflavones extract power[1]

ANALYSIS	SPECIFICATION	RESULTS
Appearance	Light yellow fine powder	Complies
Odor	Characteristic	Complies
Assay(Isoflavones, HPLC[2])	≥80.0%	82.08%
Daidzin		45.20%
Glycitin		26.26%
Genistin		8.21%
Daidzein		1.60%
Glycitein		0.51%
Genistein		0.30%
Sieve Analysis	100% pass 80 mesh	Complies
Loss on Drying	≤5.0%	1.87%
Ash	≤5.0%	1.93%
GMO	None	Complies
Heavy Metal	<10ppm	Complies
As	<1ppm	Complies
Cd	<0.5ppm	Complies
Hg	<0.1ppm	Complies
**Microbiology**		
Total Plate Count	<1000cfu/g	870cfu/g
Yeast & Mold	<100cfu/g	55cfu/g
E.Coli	Negative	Complies
Salmonella	Negative	Complies

[1]The gradient elution program of HPLC used to detect the soybean isoflavones

[2]HPLC=high performance liquid chromatography. Chromatographic conditions: Chromatographic experiments were performed with a C18 column (150 mm × 4.6 mm, 5 μm) (Shimadzu, Kyoto, Japan). Mobile phase A was water, mobile phase B was acetonitrile, the flow rate was 1.0 mL/min, the column temperature was 40°C, the injection volume was 10 μL and the detection wavelength was 260 nm.

Supplemental Figures

Supplemental Figure S1 Body measurements. 1head length, 2withers height, 3rump height, 4body length, 5chest girth, 6chest depth, 7cannon bone circumference, 8abdominal girth

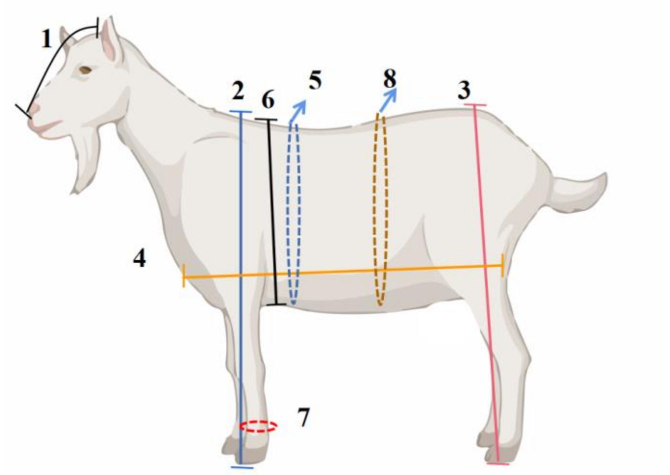

Supplement: Supplementary file 1 [file animals-14-01188-s001.zip › Supplemental material 1.pdf]
